# Supplementary material for: Asynchronous magnetic resonance elastography: Shear wave speed reconstruction using noise correlation of incoherent waves
Source: Magn Reson Med. 2022 Oct 27;89(3):990–1001. doi: 10.1002/mrm.29502 (PMC9792433; doi:10.1002/mrm.29502)
Supplement: Supplementary file 1 — DATA S1 MATLAB code used to generate the numerical simulations of Figure 1 [file MRM-89-990-s002.zip › k-Wave/helpfiles/k-wave_therapeutic_ultrasound_simulation.html]

Ultrasound Simulation (k-Wave)


# Therapeutic Ultrasound Simulation

---

- Example: Modelling a Focused Ultrasound Transducer
- Example: Calculating Heat Deposition
- Example: Beam Patterns In Heterogeneous Media
